# Spinal Autofluorescent Flavoprotein Imaging in a Rat Model of Nerve Injury-Induced Pain and the Effect of Spinal Cord Stimulation

**DOI:** 10.1371/journal.pone.0109029

**Published:** 2014-10-03

**Authors:** Joost L. M. Jongen, Helwin Smits, Tiziana Pederzani, Malik Bechakra, Mehdi Hossaini, Sebastiaan K. Koekkoek, Frank J. P. M. Huygen, Chris I. De Zeeuw, Jan C. Holstege, Elbert A. J. Joosten

**Affiliations:** 1 Dept. of Neurology, Erasmus MC, Rotterdam, the Netherlands; 2 Pain Management and Research Center, UMC+, Maastricht, the Netherlands; 3 Dept. of Neuroscience, Erasmus MC, Rotterdam, the Netherlands; 4 Dept. of Pain Medicine, Erasmus MC, Rotterdam, the Netherlands; 5 Netherlands Institute for Neuroscience, Royal Academy for Arts and Sciences, Amsterdam, the Netherlands; University of South California, United States of America

## Abstract

Nerve injury may cause neuropathic pain, which involves hyperexcitability of spinal dorsal horn neurons. The mechanisms of action of spinal cord stimulation (SCS), an established treatment for intractable neuropathic pain, are only partially understood. We used Autofluorescent Flavoprotein Imaging (AFI) to study changes in spinal dorsal horn metabolic activity. In the Seltzer model of nerve-injury induced pain, hypersensitivity was confirmed using the von Frey and hotplate test. 14 Days after nerve-injury, rats were anesthetized, a bipolar electrode was placed around the affected sciatic nerve and the spinal cord was exposed by a laminectomy at T13. AFI recordings were obtained in neuropathic rats and a control group of naïve rats following 10 seconds of electrical stimulation of the sciatic nerve at C-fiber strength, or following non-noxious palpation. Neuropathic rats were then treated with 30 minutes of SCS or sham stimulation and AFI recordings were obtained for up to 60 minutes after cessation of SCS/sham. Although AFI responses to noxious electrical stimulation were similar in neuropathic and naïve rats, only neuropathic rats demonstrated an AFI-response to palpation. Secondly, an immediate, short-lasting, but strong reduction in AFI intensity and area of excitation occurred following SCS, but not following sham stimulation. Our data confirm that AFI can be used to directly visualize changes in spinal metabolic activity following nerve injury and they imply that SCS acts through rapid modulation of nociceptive processing at the spinal level.

## Introduction

Flavoproteins are involved in a wide array of biological processes, among which adenosine triphosphate production via the mitochondrial electron transport chain. During this process the flavoprotein moieties of respiratory chain complexes I and II are oxidized, resulting in green fluorescence when illuminated with blue-spectrum light. This oxidation is followed by a reduction when the energy demand of a cell has been met, overall resulting in a bi-phasic fluorescence response. The light phase of flavoprotein autofluorescence may be used as a marker for neuronal (metabolic) activity [1]. We and others have demonstrated a linear relationship between the intensity of the neuronal stimulus and flavoprotein autofluorescence [2], [3].

Since autofluorescent flavoprotein imaging (AFI) is an optical method, it is suitable to monitor activity in superficial areas of the nervous system such as the somatosensory cortex [4]–[8], auditory cortex [9], [10], visual cortex [11], [12], cerebellar cortex [13], [14] and superficial dorsal horn of the spinal cord [3]. A major advantage is that it enables imaging of large areas at high-resolution in both the spatial (down to10×10 µm) and temporal (up to 100 frames/second) domain simultaneously. Furthermore, AFI directly represents neuronal metabolic activity, in contrast to intrinsic optical imaging [15] or fMRI using the BOLD signal [16]. AFI, however, does not allow imaging of deep structures like the deep dorsal horn of the spinal cord and has a relatively low signal-to-noise ratio [3].

Peripheral nerve injury often induces pain, which is, among others, driven by sensitization mechanisms within the spinal cord [17]. These sensitization mechanisms may be accurately monitored using autofluorescent flavoprotein imaging of the superficial spinal dorsal horn, as was shown previously using intraplantar capsaicin injection [3], [18]. The Seltzer model consists of partial ligation of the proximal part of the sciatic nerve, which generates pain behavior in rats, closely resembling the clinical condition of Complex regional Pain Syndrome (CRPS) type 2 in humans [19]. CRPS type 2 in turn has many characteristics of painful neuropathy, including spontaneous and evoked pain [20], [21]. Therefore, the Seltzer model may be considered a relevant model of nerve injury induced pain [22].

Painful neuropathy and CRPS are frequently refractory to pharmacological treatment and physical therapy. Spinal cord stimulation (SCS) is a generally accepted therapy in patients with CRPS [23]–[25] and recently SCS has yielded promising results in patients with painful diabetic neuropathy [26], [27]. SCS is based on the gate-control theory from the 1960's [28], although the exact mechanism of action is still only partially clarified [29]. Probably, GABA-ergic interneurons, situated in the substantia gelatinosa, are of major importance in SCS treatment of chronic neuropathic pain [30]. It should be stressed, however, that the latter evidence is based on data obtained after dialysis of the spinal dorsal horn [31] or immunohistochemical visualization [32]. Hence, these data present only indirect evidence on the exact spatial and temporal changes of SCS in the spinal superficial dorsal horn.

We first set out experiments to study the mechanisms of sensitization in the superficial spinal dorsal horn by applying AFI to the Seltzer model. Subsequently, changes in nociceptive transmission in the superficial dorsal horn of chronic neuropathic rats brought about by SCS were visualized at a high spatial and temporal resolution using the same AFI imaging technology.

## Materials and Methods

### Animal preparation

All animal experimentation conformed to the guidelines laid out in the Guide for the Care and Use of Laboratory Animals (National Academy of Sciences) and was approved by the Institutional Animal Ethics Committee of Erasmus MC Rotterdam (EMCnr. 115-08-26). Recordings were obtained from a total number of 18 young adult male Sprague Dawley rats from Harlan or Charles River, the Netherlands, weighing 250–300 g. Neuropathic pain was induced by partial ligation of the sciatic nerve as described by Seltzer et al [19]. Recordings from 20 Wistar rats with similar age/weight from previous experiments [3] were used as controls.

### Behavioral tests

Behavioral testing took place before the Seltzer operation and at post-operative days 10, 12 and 14. Every time before behavioral testing, rats were habituated to the experimenter (T.P. or M.B.), the room in which the behavioral experiments took place and the transparent chamber used for von Frey testing, for at least half an hour. Mechanical sensitivity was assessed by testing the withdrawal response to increasing in thickness von Frey filaments (Stoelting Co., Wood Dale, IL). The threshold was set at three out of five withdrawal responses. After testing for mechanical sensitivity, thermal thresholds were assessed by the hotplate test. The surface of the hot plate was heated to a constant temperature of 51°C. Rats were placed on the hot plate (25.4 cm×25.4 cm) (Ugo Basile Srl., Comerio, VA, Italy), which was surrounded by a transparent plexiglas chamber with an open top, and the latency to respond with either a hind paw lick or hind paw flick was measured. Immediately after a response rats were removed from the hotplate. Rats were also removed if they did not respond after 30 seconds, to prevent tissue injury.

### Autofluorescent Flavoprotein Imaging in rats with nerve injury

After behavioral testing at day 14, rats were anesthetized and surgery and image acquisition for autofluorescent flavoprotein imaging of the spinal cord was performed as previously described [3], using a high speed 16-bit CCD camera with 512×512 pixel resolution (Roper Scientific, Evry, France). A silicon cuff containing a bipolar electrode was placed around the left sciatic nerve proximal to the knee, i.e. just distal to the suture from the partial nerve ligation. As a measure of fluorescence, generally ΔF/F is used. ΔF/F represents the change in fluorescence intensity of each pixel during registration relative to the mean fluorescence intensity of these pixels in frames preceding electrical stimulation (see also [3]). AFI responses were expressed as the maximal ΔF/F change in fluorescence following stimulation (AFI intensity), or as the area with an AFI intensity above a predefined ΔF/F level (area of excitation). This predefined ΔF/F level was always kept constant. Recordings using 2.5 mA, 10 Hz electrical stimulation of the left sciatic nerve lasting 10 seconds were obtained in 13 Sprague Dawley rats that had undergone partial sciatic nerve ligation and compared with recordings from 20 naïve Wistar rats from previous experiments [3], using the same electrical stimulus. A similar experiment was carried out in rats (*n* = 5+5) using a 10 seconds lasting 1 Hz innocuous palpation of the plantar surface of the left hind paw [3].

### Autofluorescent Flavoprotein Imaging in rats with nerve injury undergoing SCS or sham stimulation

Following “before treatment” AFI recordings in neuropathic Sprague Dawley rats (see above), a monopolar stimulation system with a 3.0×1.0×0.1 mm platinum-iridium rectangular plate micro cathode was placed in the dorsal epidural space at the T12-T13 vertebral level, while the anode was placed in a subcutaneous pocket on the back, and rats underwent 50 Hz, amplitude 2/3 of motor threshold SCS (*n* = 7) or no electrical stimulation (sham; *n* = 6) for 30 minutes, as previously described [33]. Immediately following SCS or sham the micro cathode was removed and AFI responses to left sciatic nerve electrical stimulation (same stimulus as baseline) were recorded at T = 0 after SCS or sham and then every 5 minutes for up to an hour. Both the intensity of the AFI response (expressed as ΔF/F of the light phase) and the area with an AFI intensity above a predefined ΔF/F level was calculated and expressed as a percentage of ΔF/F before treatment. At the end of the experiment, rats were euthanized with an overdose of intraperitoneal urethane.

### Statistical analysis and presentation of the figures

Statistical analyses were performed using GraphPad Prism version 6.0e and SPSS statistics version 21 software. For a comparison of means of behavioral responses, a repeated-measures ANOVA was used. For an overall comparison of means of AFI intensities and areas of excitation between naïve and neuropathic rats and between sides, two-way ANOVAs were used. For a comparison of means of AFI intensities following non-noxious palpation in naïve and neuropathic rats, an unpaired *t*-test was used. For comparing the effect of SCS versus sham on AFI intensities and area of excitation, paired *t*-tests were used, Pearson's correlation coefficients were calculated and a linear regression analysis was performed. The data in the figures are expressed as mean ± SEM. Figures were composed in Photoshop CS6 software version 13.0.6. Adjustments were made only to brightness and contrast and applied evenly to all panels of a figure.

## Results

**Figure 1:**
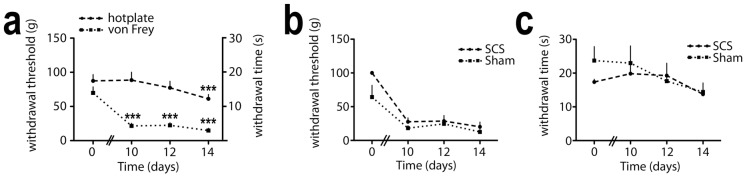
Behavioral data of rats that underwent partial ligation of the proximal sciatic nerve (Seltzer model). (A) Combined results of the von Frey withdrawal thresholds and hotplate latencies, at baseline and 10, 12 and 14 days after nerve ligation, from al 18 rats in the study, demonstrating tactile and thermal hyperalgesia. Error bars indicate SEM. *p*<0.01; repeated measures ANOVAs, source of variation timepoint; ***p<0.01; pairwise comparisons of day 0 versus day 10, 12 and 14, using Bonferroni correction. (B,C) Von Frey withdrawal thresholds (B) and hotplate latencies (C) from 7 neuropathic rats that subsequently underwent SCS and 6 neuropathic rats that subsequently underwent sham stimulation, demonstrating a similar degree of tactile and thermal hyperalgesia in both groups. Error bars indicate SEM. *p*>0.06; repeated measures ANOVAs, source of variation treatment.

Following partial ligation of the left sciatic nerve in the thigh, all 18 Sprague Dawley rats used in this study developed mechanical and thermal hypersensitivity characteristic of the Seltzer model (Table S1) [19]. Repeated-measures ANOVAs (source of variation timepoint) demonstrated that the decrease in von Frey thresholds and hotplate latencies was statistically significant (Fig. 1A; *p*<0.01).

**Figure 2:**
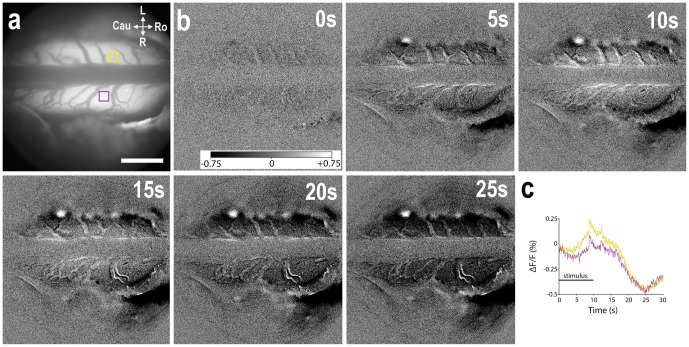
Spinal cord AFI signal following nociceptive electrical stimulation of the sciatic nerve, in a rat with partial ligation of the proximal sciatic nerve (Seltzer model). (A) Image of background fluorescence showing the dorsal surface of the spinal cord at the T13 vertebral level. The upper half is left, the lower half is right, the dark structure in the center is a dural vein. (B) Subtracted ΔF/F images at various time points after start of electrical stimulation (2.5 mA, 10 Hz) of the left sciatic nerve. (C) Graph showing the time course of ΔF/F in the yellow (left, i.e. ipsilateral or stimulated side) and purple (right, i.e. contralateral side) 20×20 pixel square selections in (A). Scale bar, 1 mm. Gray scale bar ranging from −0.75% (black) to +0.75% (white) of the 16-bit range; Cau  =  caudal, Ro  =  rostral, L  =  left, R  =  right.

We then set out to capture AFI responses in these neuropathic Sprague Dawley rats. A typical AFI recording of a 10 s, 2.5 mA, 10 Hz electrical stimulation of the left sciatic nerve showed a steep increase in spinal fluorescence (light phase) immediately after the start of the stimulation, followed by a decrease below baseline (dark phase) (Fig. 2; Movie S1). This pattern of activity is typical of autofluorescent flavoprotein imaging in the brain and spinal cord. In the rest of this paper we only use the light phase for analysis, since this is the default measure of activity in AFI.

**Figure 3:**
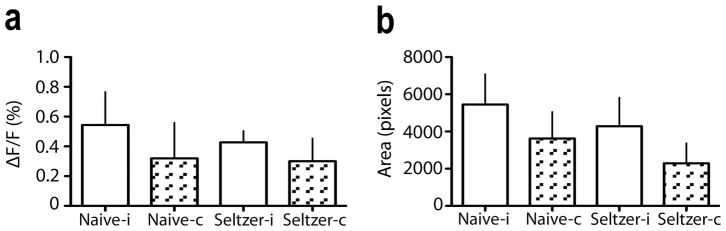
Mean intensity of the AFI signal (A) and area of excitation (B) following nociceptive electrical stimulation of the sciatic nerve, in naïve versus neuropathic rats (Seltzer model), on the ipsilateral (i) and contralateral (c) side of the nerve injury and nerve stimulation. Error bars indicate SEM; *n* = 20 naïve rats, *n* = 13 neuropathic rats.

Next, we compared mean AFI intensities and areas of excitation following 10 s, 2.5 mA, 10 Hz electrical stimulation of 13 Sprague Dawley rats with partial nerve ligation, with those from 20 naïve Wistar rats from a previous study [3], that had undergone exactly the same electrical stimulation protocol (Fig. 3; Table S2). The main effects of both type of animal (naïve Wistar versus neuropathic Sprague-Dawley rats) and side (ipsilateral versus contralateral) on AFI intensity and area of excitation were not significantly different, nor were the interactions between type of animal and side on AFI intensity and area of excitation (*p*>0.23; two-way ANOVAs).

**Figure 4:**
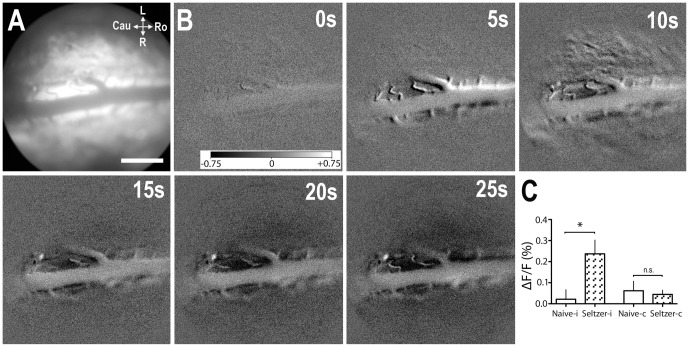
Intensity of the AFI signal, following innocuous palpation in naïve rats and rats with partial ligation of the proximal sciatic nerve (Seltzer model). (A) Image of background fluorescence of the dorsal surface of the spinal cord at T13. (B) Subtracted ΔF/F images at various time points after start of 10 seconds, 1 Hz innocuous palpation of the plantar surface of the left hindpaw. (C) Mean ΔF/F of the light phase in 20×20 pixel square selections on the ipsi-(i) and contralateral (c) side at the L4-6 spinal level, in naïve rats from our previous experiments [3] and in rats with partial ligation of the proximal sciatic nerve (Seltzer model). Scale bar, 1 mm. Gray scale bar ranging from −0.75% (black) to +0.75% (white) of the 16-bit range. Error bars indicate SEM; *p<0.05; unpaired *t*-test; *n* = 5 naïve rats, *n* = 5 neuropathic rats.

Since it is known [17], [19] that in neuropathic pain states also innocuous stimuli may elicit nociceptive activity in the superficial dorsal horn, we investigated AFI responses to 10 s, 1 Hz innocuous palpation of the left hind paw. We compared AFI intensities in 5 neuropathic Sprague Dawley rats with those from 5 naïve Wistar rats from our previous study (Fig. 4; Movie S2, Table S3). While we have demonstrated that after innocuous palpation in naïve rats AFI intensity is not different from recordings without stimulation, there was a robust increase in AFI intensity following palpation in neuropathic rats on the ipsilateral side, which was statistically significantly different from naïve rats (*p* = 0.03; unpaired *t*-test). Results on the contralateral side of naïve and neuropathic rats were not statistically significantly different (*p* = 0.7; unpaired *t*-test).

**Figure 5:**
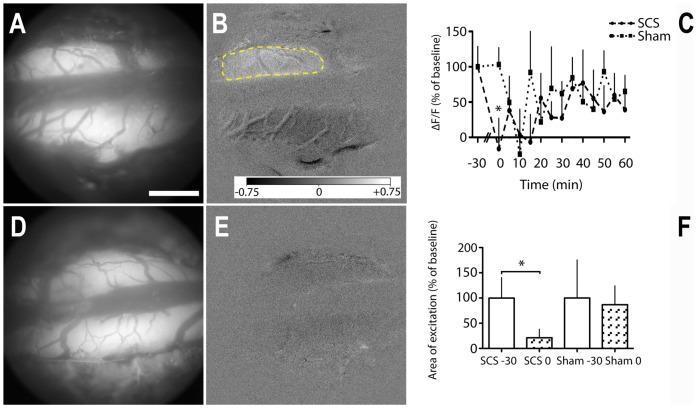
Effect of 30 minutes SCS or sham stimulation on the intensity of the AFI signal and area of excitation in response to sciatic nerve electrical stimulation, in rats with partial ligation of the proximal sciatic nerve (Seltzer model). (A,D) Images of background fluorescence of the dorsal surface of the spinal cord at T13 of a sham (A) and SCS treated rat (D). (B,E) Area of excitation (yellow) on the ipsilateral side, directly after sham stimulation (B); after SCS, in this rat, there is no area exceeding the predefined ΔF/F level (E). (C) Time course of the intensity of the AFI signal after SCS or sham stimulation (T = 0 min), as a percentage of ΔF/F before treatment (T = -30 min), in 20×20 pixel square selections on the ipsilateral side at the L4-L6 spinal level. (F) Mean areas of excitation on the ipsilateral side directly after SCS or sham stimulation (T = 0 min), as a percentage of the areas before treatment (T = -30 min). Scale bar, 1 mm; Grayscale bar ranging from −0.75% (black) to +0.75% (white) of the 16-bit range; Error bars indicate SEM; *p<0.05; paired *t*-tests; *n* = 7 SCS, *n* = 6 sham stimulation.

Finally, the effect of spinal cord stimulation on AFI responses in neuropathic Sprague Dawley rats was investigated. Prior to stimulation, neuropathic pain behavior was not statistically significantly different between rats that underwent SCS (*n* = 7) or sham (*n* = 6) stimulation (Fig. 1B and 1C; *p*>0.06; repeated-measures ANOVA, source of variation treatment). We then studied relative AFI responses, expressed as a percentage of the “before treatment” AFI response, in 7 neuropathic rats after 30 minutes 50 Hz spinal cord stimulation, using a platinum cathode at the T12-T13 vertebral level, and in 6 neuropathic rats that underwent sham stimulation, i.e. with cathode placement but without the 50 Hz electrical stimulus (Table S4). In rats with SCS there was a strong and statistically significant reduction in AFI intensity as well as area of activation directly after cessation of SCS on the ipsilateral side (Fig. 5; *p* = 0.049 and *p* = 0.041 respectively; paired *t*-test), while in rats that underwent sham stimulation there was no statistically significant reduction (*p*>0.8; paired *t*-test). In the period from T = 0 to T = 60 minutes following cessation of SCS, there was a statistically significant linear increase in AFI intensity on the ipsilateral side in the rats with SCS (slope 0.92%ΔF/F *min^-^1; *p* = 0.021; Pearson's correlation coefficient and linear regression analysis), indicating a reducing efficacy of SCS at these later time-points. In the rats with sham stimulation the slope was not statistically significant non-zero (slope 0.19%ΔF/F *min^-^1; *p* = 0.72; Pearson's correlation coefficient and linear regression analysis), indicating no treatment effect in the rats that underwent sham stimulation.

## Discussion

Nerve injury-induced pain is a complex disorder, which is driven by a multitude of plastic changes, like sensitization of (peripheral) nociceptors [34], [35], increased excitability of spinal cord projection neurons [36], decreased propriospinal [37] and descending [38] spinal inhibition, spinal glia activation [39] and changes in the transmission of nociceptive signals in the brainstem and neocortex [40]. In this study we focused on changes following nerve injury in (metabolic) activity in the superficial dorsal horn, a major relay station in the transmission of the nociceptive signal to higher brain centers. Using the Seltzer model of nerve injury-induced pain and AFI, we first demonstrate that although neuropathic rats did not have an increased activation following nociceptive electrical stimulation compared to naïve rats, they express a robust ipsilateral response to non-noxious palpation, which is not present in naïve rats. Secondly, we used AFI to study the effect of spinal cord stimulation on nociceptive activity in the superficial dorsal horn in neuropathic animals. AFI shows an immediate and pronounced, but short-lasting reduction in intensity and area of spinal nociceptive activity following SCS, which was not observed following sham stimulation.

We have previously put forward, that the spinal cord AFI response following primary afferent stimulation is generated by projection neurons and local interneurons in the superficial laminae of the spinal dorsal horn [16]. Secondly, we have shown that spinal AFI is suitable to study plastic changes in this area following an intraplantar capsaicin injection [3]. In this study we have used a similar approach to study changes in spinal nociceptive activity following nerve-injury. Behavioral studies in experimental animals [19], [41] and psychophysical studies [42], [43] in humans with nerve injury consistently demonstrate pain (behavior) evoked by stimuli that are not painful under normal conditions, e.g. tactile allodynia. Similarly, in the Seltzer model of nerve injury-induced pain we now demonstrate a strong ipsilateral AFI response to innocuous palpation, which was not present in naïve animals.

There were no statistically significant differences between naïve and neuropathic rats following a nociceptive 2.5 mA electrical stimulus. Although hyperalgesia to nociceptive stimuli does exist both in experimental and clinical neuropathic conditions, a strong enough electrical stimulus may saturate metabolic activity of superficial spinal dorsal horn neurons, i.e. the AFI signal. The electrical stimulus intensity that we used here is almost three times C-fiber threshold and generates a response that is close to the maximal AFI intensity that we found previously in naïve animals [3]. Although this response could be further enhanced in the acute situation by intraplantar capsaicin injection [3], the same may not be true in chronic neuropathy. Similarly, c-Fos expression, another marker of spinal nociceptive activity, is not increased in animals with chronic neuropathy compared to naïve animals, following nociceptive stimulation [44].

To reduce the number of experimental animals, we used naïve rats from a previous study [3] as controls. These animals were Wistar rats, i.e. not the same strain as the Sprague-Dawley rats that were used here because of the Seltzer model. One may therefore argue that the above-described lack of a difference in AFI activity following nociceptive electrical stimulation between naïve and neuropathic rats could be the result of a genetic difference in sensitivity to nociceptive stimuli. However, at least behaviorally Sprague-Dawley rats demonstrate a hyperalgesic phenotype in comparison to other rat strains, including Wistar rats [45], [46]. In addition, it is highly unlikely that non-noxious palpation would induce an AFI response in naïve Sprague-Dawley rats (as opposed to Wistar rats), since metabolic activity solely in the deep dorsal horn cannot be visualized by AFI. We therefore conclude that the strain differences in our study do not affect our conclusions regarding spinal nociceptive processing in nerve injury-induced pain.

Regarding our second aim, to study mechanisms of action of SCS, this is the first report directly demonstrating reduced activity in the superficial dorsal horn *in vivo* following SCS. We used the AFI response to a 2.5 mA electrical stimulus as outcome measure, since in our hands this stimulus generates the most robust and consistent AFI responses. Others have also used nociceptive stimulation to study the effect of SCS [47].

Previous studies measuring peptides involved in antinociception [31], [48] or using pharmacological approaches [49], [50] present only indirect evidence of reduced spinal nociceptive activity. Furthermore, studies of electrophysiological activity in wide dynamic range neurons in the deep dorsal horn [51] focus on an area that may not be decisive in generating the neuropathic pain phenotype [52], [53] and that may not be the locus of “gate control”, which instead is postulated to be the substantia gelatinosa in the superficial dorsal horn [28]. Our finding of decreased activity in the superficial dorsal horn is in line with two reports [54], [55] demonstrating a significant increase in c-Fos expression in the superficial dorsal horn following SCS in rats with nerve injury, which was larger than the increase in the deep dorsal horn. These c-Fos expressing neurons presumably represent inhibitory interneurons [37], considering the decrease in neuronal metabolic activity in the superficial dorsal horn. Indeed, a double immunohistochemical staining procedure revealed the presence of c-Fos positive GABA-immunoreactive neurons in the superficial dorsal horn of SCS-treated chronic neuropathic rats [32]. The latter report and that of Cui et al. [31] stress the role of GABA-ergic interneurons in the mechanism underlying SCS in chronic neuropathic pain. Nevertheless, so far no direct changes in the spatial and temporal domain related to the effect of SCS on nociceptive transmission in the superficial dorsal horn of chronic neuropathic rats have been studied. Our findings therefore provide the first direct evidence that SCS acts through modulation of nociceptive processing at the spinal segmental level.

The effect of SCS on nociceptive activity in the superficial dorsal horn that we describe here is rather short lasting, as demonstrated by a linear decrease of efficacy from SCS directly following cessation of stimulation (i.e. T = 0 min) and a lack of statistical significance between SCS and sham animals at time-points T = 5 minutes or later after SCS. A lack of statistical significance at those later time-points may be caused by a relatively low signal-to-noise ratio and technical challenges of spinal cord AFI that were discussed previously [3], resulting in large variation between recordings within the same animal and between animals. However, behavioral effects of SCS also do not outlast the duration of SCS [33]. The relatively short duration of an initially significant effect of SCS does not preclude a clinical meaningful effect of SCS in patients with nerve injury-induced pain or CRPS, since in patients spinal cord stimulators deliver continuous stimulation. Continuous stimulation during AFI recording was not feasible due to our experimental setup, as the spinal electrode prevented imaging of the spinal cord.

In conclusion, we demonstrated changes in neuronal metabolic activity in the superficial dorsal horn following nerve injury, which may reflect mechanisms of hyperalgesia in patients with neuropathic pain syndromes. Secondly, our study provides a rationale for spinal cord stimulation in neuropathic pain patients.

## Supporting Information

Movie S1 **Typical AFI recording in a neuropathic rat, following nociceptive electrical stimulation.** The video shows a stack of subtracted ΔF/F images of the dorsal surface of the spinal cord at the T13 vertebral level, before, during and after nociceptive electrical stimulation of the left sciatic nerve.
(M4V)

Movie S2 **Typical AFI recording in a neuropathic rat, following innocuous palpation.** The video shows a stack of subtracted ΔF/F images of the dorsal surface of the spinal cord at the T13 vertebral level, before, during and after innocuous palpation of the left hindpaw.
(M4V)

Table S1 **Behavioral data of neuropathic Sprague-Dawley rats.** VF0 = Von Frey threshold at day 0, etc.; HP0 = Hotplate at day 0 etc.; SCS = rats undergoing spinal cord stimulation; sham = rats undergoing sham stimulation; palpation = rats undergoing innocuous palpation.
(XLSX)

Table S2 **AFI intensity and AFI area of naïve Wistar and neuropathic Sprague-Dawley (SD) rats on the ipsilateral (i.e. left) and contralateral (i.e. right) side of the spinal cord, following nociceptive electrical stimulation of the left sciatic nerve.**
(XLSX)

Table S3 **AFI intensity of naïve Wistar and neuropathic (i.e. Seltzer) Sprague-Dawley (SD) rats on the ipsilateral (ipsi) and contralateral (contra) side of the spinal cord, following innocuous palpation of the left hindpaw.**
(XLSX)

Table S4 **AFI intensities and AFI areas following spinal cord versus sham stimulation.** SCS vs Sham deltaFF: AFI intensities of 7 SCS and 6 Sham rats as a percentage of mean ΔF/F before treatment, up to one hour (T = 60) following cessation of SCS or Sham; SCS and Sham deltaFF bf vs T0: AFI intensities of 7 SCS and 6 Sham rats as a percentage of mean ΔF/F before treatment, before and directly following cessation (T = 0) of SCS of Sham; SCS and Sham area% bef vs T = 0: AFI areas of 7 SCS and 6 Sham rats as a percentage of mean area before treatment, before and directly following cessation (T = 0) of SCS or Sham; Linear regression = mean relative AFI intensities of 7 SCS and 6 Sham rats, from directly following cessation of SCS or Sham (T = 0) to one hour (T = 60).
(XLSX)
